# Supplementary material for: Competition for resources can promote the divergence of social learning phenotypes
Source: Proc Biol Sci. 2020 Feb 19;287(1921):20192770. doi: 10.1098/rspb.2019.2770 (PMC7062025; doi:10.1098/rspb.2019.2770)
Supplement: Supplementary Materials [file rspb20192770supp1.pdf]

Gilman R.T., Johnson F., Smolla M. 2020. Competition for resources can promote the divergence of social learning phenotypes. *Proceedings of the Royal Society B: Biological Sciences* 10.1098/rspb.2019.2770.

Supplementary Information

S1. Constructing the transition rate matrix $\mathbf{M}(\mathbf{v})$

Let $\mathbf{M}(\mathbf{v})$ be the transition rate matrix for the probability vector $\mathbf{v}$. Entry $m_{ij}(\mathbf{v})$ of $\mathbf{M}(\mathbf{v})$ describes the rate at which patches transition from state j to state i in a system occupied by a monomorphic forager population at density D with a social learning rule described by the vector $\mathbf{s_r}$. Entry s_{rk} of $\mathbf{s_r}$ describes the probability that a forager in a patch in state k learns socially, and the states governed by $\mathbf{s_r}$ are the states described by $\mathbf{v}$. In the basic model the probability of social learning is the same in all patches (*i.e.*, $s_{rk} = s_r$ for all k), but in the model extensions the probability of social learning depends on the patch quality and/or occupancy. The state of a patch changes when i) the patch quality changes, ii) a forager departs, or iii) a forager arrives. Thus, for $j \neq i$,

$$m_{ij}(\mathbf{v}) = m_{ijc} + m_{ijd}(\mathbf{v}) + m_{ija}(\mathbf{v}) \qquad \text{(S1)}$$

where m_{ijc} is the rate at which patches transition from state j to state i due to changes in patch quality, $m_{ijd}(\mathbf{v})$ is the rate at which patches transition from state j to state i due to the departure of a forager, and $m_{ija}(\mathbf{v})$ is the rate at which patches transition from state j to state i due to the arrival of a forager. Moreover,

$$m_{jj}(\mathbf{v}) = -\sum_{i \neq j} m_{ij}(\mathbf{v}). \qquad \text{(S2)}$$

Due to change events, bad patches become good at rate cg and good patches become bad at rate c(1-g). Thus,

$$m_{ijc} = cg \tag{S3}$$

for $i \in \{1, 2, \ldots, 1 + n_{max}\}$ and $j = i + 1 + n_{max}$, and

$$m_{ijc} = c(1 - g) \tag{S4}$$

for $j \in \{1, 2, \ldots, 1 + n_{max}\}$ and $i = j + 1 + n_{max}$. Otherwise, $m_{ijc} = 0$.

The rate at which patches in state j lose foragers due to learning is equal to the number of foragers in the patch times the rate at which foragers learn, l, times the probability that learning discovers a patch in a state better than j. Let $\mathbf{p}$ be the number of foragers in patches in each state tracked by $\mathbf{v}$. Thus, $p_i = i - 1$ for $i \in \{1, 2, \ldots, 1 + n_{max}\}$ and $p_i = i - (2 + n_{max})$ for $i \in \{2 + n_{max}, 3 + n_{max}, \ldots, 2 + 2\, n_{max}\}$. Then,

$$m_{ijd}(\mathbf{v}) = p_j l \left(s_{rj} \sum_{k=1}^{N} \frac{p_k v_k}{D} + \left(1 - s_{rj}\right) \sum_{k=1}^{N} v_k \right) \tag{S5}$$

for $j \in \{3, 4, \ldots, 2 + 2\, n_{max}\}$, $i = j - 1$, and $N = \min(j - 2, n_{max})$. Otherwise, $m_{ijd}(\mathbf{v}) = 0$. The two terms in the outer parentheses in equation S5 capture the probability that a forager learns about a patch better than its current patch by social learning or by individual learning, respectively. The upper limit of the summation captures the fact that foragers from good patches move only to

patches with fewer competitors, but foragers from bad patches will move to any available good patch.

The rate at which foragers arrive in patches in state *j* can be decomposed into two parts: the rate at which foragers arrive after social learning, $m_{ijas}(\mathbf{v})$, and the rate at which foragers arrive after individual learning, $m_{ijai}(\mathbf{v})$. Thus,

$$m_{ija}(\mathbf{v}) = m_{ijas}(\mathbf{v}) + m_{ijai}(\mathbf{v}) \quad \text{(S6)}$$

The rate at which patches in state *j* gain foragers due to social learning is equal to the density of foragers in patches in states worse than *j*, times the rate at which those foragers learn socially, times the relative rate at which patches in state *j* are discovered by social learning. Thus,

$$m_{ijas}(\mathbf{v}) = \left(\sum_{k=j+2}^{2+2n_{max}} p_k v_k l s_{rk} \right) \frac{p_j}{D} \quad \text{(S7)}$$

for $j \in \{1, 2, \ldots, n_{max}\}$ and $i = j + 1$, and $m_{ijas}(\mathbf{v}) = 0$ otherwise. The rate at which patches in state *j* gain foragers due to individual learning is equal to the density of foragers in patches in states worse than *j* times the rate at which those foragers learn individually. The relative rate of discovering patches in state *j* is omitted because foragers that learn individually are equally likely to discover any patch. Thus,

$$m_{ijai}(\mathbf{v}) = \sum_{k=j+2}^{2+2n_{max}} p_k v_k l(1 - s_{rk}) \quad \text{(S8)}$$

for $j \in \{1, 2, \ldots, n_{max}\}$ and $i = j + 1$, and $m_{ijai}(\mathbf{v}) = 0$ otherwise. Equations (S7) and (S8) do not include transitions from state $j = 1 + n_{max}$ because these patches are fully occupied and cannot be joined, or from states $j \in \{2 + n_{max}, 3 + n_{max}, \ldots\}$ because these patches offer no resources and so are never joined.

S2. Constructing the transition rate matrix **N**

Let **N** be the transition rate matrix for the vector **u**. Thus, entry n_{ij} of **N** describes the rate at which a rare mutant forager with a social learning rule $\mathbf{s_m}$ and occupying a patch in state *j* moves to a patch in state *i* when the resident population is at the steady state distribution described by **v***. Transitions occur when i) the quality of a mutant's patch changes, ii) a resident leaves a patch occupied by a mutant, iii) a resident joins a patch occupied by a mutant, or iv) a mutant moves between patches. Thus,

$$n_{ij} = n_{ijc} + n_{ijd} + n_{ija} + n_{ijm} \tag{S9}$$

where n_{ijc} captures transitions due to changes in patch quality, n_{ijd} captures transitions due to the departure of residents, n_{ija} captures transitions due to the arrival of residents, and n_{ijm} captures transitions due to the movement of mutants. Moreover,

$$n_{jj} = -\sum_{i \neq j} n_{ij}. \tag{S10}$$

Due to change events, bad patches become good at rate cg and good patches become bad at rate $c(1\text{-}g)$. Thus,

$$n_{ijc} = cg \tag{S11}$$

for $i \in \{1,2, \dots 1 + n_{max}\}$ and $j = i + 1 + n_{max}$, and

$$n_{ijc} = c(1 - g) \tag{S12}$$

for $j \in \{1,2, \dots 1 + n_{max}\}$ and $i = j + 1 + n_{max}$. Otherwise, $n_{ijc} = 0$.

The rate at which residents leave patches occupied by mutants is equal to the number of residents in the patch times the rate at which those residents learn about patches better than those they currently occupy. Thus,

$$n_{ijd} = p_j l \left(s_{rj} \sum_{k=1}^{j-1} \frac{p_k v_k^*}{D} + \left(1 - s_{rj}\right) \sum_{k=1}^{j-1} v_k^* \right) \tag{S13}$$

for $j \in \{2, 3, \dots, n_{max}\}$ and $i = j - 1$, and

$$n_{ijd} = p_j l \left(s_{rj} \sum_{k=1}^{n_{\max}} \frac{p_k v_k^*}{D} + \left(1 - s_{rj}\right) \sum_{k=1}^{n_{\max}} v_k^* \right) \tag{S14}$$

for $j \in \{2 + n_{max}, 3 + n_{max}, \dots, 2\, n_{max} + 1\}$ and $i = j - 1$. Otherwise, $n_{ijd} = 0$. In equations (S13) and (S14), the two terms in the outer parentheses account for residents that learn about patches better than their current patch by social learning and by individual learning, respectively. Equation (S13) accounts for residents from good patches, which move only to less-occupied good patches. Equation (S14) accounts for residents from bad patches, which will move to any available good patch. There are no transitions from states $j \in \{1, 2 + n_{max}\}$ because there are no resident foragers in those patches, and there are no transitions from states $j \in \{1 + n_{max}, 2 + 2\, n_{max}\}$ because there are no mutants in those patches.

The rate at which resident foragers arrive in patches occupied by mutants can be decomposed into two parts: the rate at which residents arrive

after social learning, n_{ijas}, and the rate at which residents arrive after individual learning, n_{ijai}. Thus,

$$n_{ija} = n_{ijas} + n_{ijai} \tag{S15}$$

The rate at which residents join mutants in state *j* after social learning is equal to the density of residents in patches in states worse than *j*, times the rate at which those residents learn socially, times the relative rate at which patches in state *j* are discovered by social learning. Thus,

$$n_{ijas} = \left(\sum_{k=j+3}^{2+2n_{\max}} p_k v_k^* l s_{rk} \right) \frac{1+p_j}{D} \tag{S16}$$

for $j \in \{1, 2, ..., n_{max} - 1\}$ and $i = j + 1$, and $n_{ijas} = 0$ otherwise. The rate at which residents join mutants in state *j* after individual learning is equal to the density of residents in patches in states worse than *j*, times the rate at which residents learn individually. The relative rate at which patches in state *j* are discovered is omitted because individual learning is equally likely to discover any patch. Thus,

$$n_{ijai} = \sum_{k=j+3}^{2+2n_{\max}} p_k v_k^* l \left(1 - s_{rk}\right) \tag{S17}$$

for $j \in \{1, 2, ..., n_{max} - 1\}$ and $i = j + 1$, and $n_{ijai} = 0$ otherwise. Equations (S16) and (S17) do not include transitions from states $j \in \{n_{max}, n_{max} + 1\}$ because these patches are fully occupied and cannot be joined, or from states $j \in \{2 + n_{max}, 3 + n_{max}, ...\}$ because these patches offer no resources and are never joined.

A mutant moves from one patch to another if it learns about a patch better than the one it currently occupies. If state *i* is better than state *j*, then the rate at which mutants move from patches in state *j* to patches in state *i* is equal to the rate at which they learn about those patches. Otherwise, it is zero. Thus,

$$n_{ijm} = l\left(s_{mj}\frac{p_j v_j^*}{D} + \left(1 - s_{mj}\right)v_j^*\right) \qquad \text{(S18)}$$

for $j \in \{2, \ldots, 2 + 2\, n_{max}\}$ and $i \leq \min(j - 1, n_{max})$, and $n_{ijm} = 0$ otherwise. There are no transitions from state $j = 1$ because mutants never leave good patches if they are alone. There are no transitions to state $i = n_{max} + 1$ because mutants cannot enter patches with n_{max} residents, or to states $i > n_{max} + 1$ because mutants never move to patches with no resources.

S3. Illustrating the mechanisms that favour the divergence of social learning phenotypes in the basic model

Consider a population that is monomorphic for the convergence-stable social learning phenotype. Social learning phenotypes are favoured to diverge if rare mutants that expresses phenotypes different from the convergence-stable phenotype can collect more resources than foragers that expresses the convergence-stable phenotype. Here, we explain why this happens under some conditions but not others. We begin with a set of useful definitions, and then we explain the mechanisms that promote the divergence of social learning in the basic model.

Preliminaries

Let $\mathbf{u^*}(s)$ be the probability vector that describes the steady-state distribution of a rare forager expressing social learning phenotype s in a population otherwise monomorphic for the convergence-stable social learning phenotype s^*. In particular, entry i of $\mathbf{u^*}(s)$ is the proportion of foragers in good patches with i occupants for $i \in \{1, 2, \ldots, 1 + n_{max}\}$, and the proportion of foragers in bad patches with $i - (n_{max} + 1)$ occupants for $i \in \{2 + n_{max}, 3 + n_{max}, \ldots, 2 + 2\, n_{max}\}$. We compute $\mathbf{u^*}(s)$ by numerically solving equation (4) in the body of the paper.

Let $\mathbf{L_{sl}}$ be the transition matrix in which entry $L_{sl,ij}$ describes the probability that a forager moves from a patch in state j to a patch in state i after a social learning event. Similarly, let $\mathbf{L_{il}}$ be the transition matrix in which entry $L_{il,ij}$

describes the probability that a forager moves from a patch in state *j* to a patch in state *i* after an individual learning event. The entries of $\mathbf{L_{sl}}$ are

$$L_{sl,ij} = \begin{cases} \frac{(i-1)v_i^*}{D}, & i \leq Z \\ 1 - \sum_{k=1}^{Z} \frac{(k-1)v_k^*}{D}, & i = j \\ 0, & \text{otherwise} \end{cases} \tag{S20}$$

and the entries of $\mathbf{L_{il}}$ are

$$L_{il,ij} = \begin{cases} v_i^*, & i \leq Z \\ 1 - \sum_{k=1}^{Z} v_k^*, & i = j \\ 0, & \text{otherwise} \end{cases}, \tag{S21}$$

where v_i^* is the *i*th entry of the vector $\mathbf{v^*}$ defined in the body of the paper, $Z = \min(j - 1, n_{max})$, and *D* is the population density. Let $\mathbf{L}(s)$ be the transition rate matrix due to a single learning event for a forager with social learning rate *s*. Then, for the basic model,

$$\mathbf{L}(s) = s\,\mathbf{L_{sl}} + (1 - s)\,\mathbf{L_{il}}. \tag{S22}$$

Let ϕ_i be the expected excess resources that a forager expressing the convergence-stable social learning phenotype will collect if it occupies a patch in state *i*, compared to the average resources collected by foragers in the system at the steady state. Thus, ϕ_i measures the long-term value of a patch in state *i* to foragers that occupy that patch. Over the long term, the resources a forager will collect if it occupies a particular patch depend not only on the quality of the patch and the number of foragers in the patch, but also on the rate at which patch qualities change, the rate at which other foragers join or leave the patch,

and how soon the forager can expect to find a better patch. We define the matrix $\mathbf{U}(t)$ in which entry $U_{ij}(t)$ is the proportion of foragers that occupied a patch in state j at time 0 and occupy a patch in state i at time t. Thus, $\mathbf{U}(t)$ is the solution to

$$\frac{d\mathbf{U}(t)}{dt} = \mathbf{NU}(t) \tag{S23}$$

where $\mathbf{N}$ is computed as in supplementary materials S2. As $t \rightarrow \infty$, the columns of $\mathbf{U}(t)$ approach $\mathbf{u^*}(s^*)$. Let $\mathbf{U^*}$ be the square matrix with columns $\mathbf{u^*}(s^*)$ and let $\boldsymbol{\rho}$ be the vector of resource collection rates that foragers achieve in each patch state (*i.e.*, $\rho_i = i^{-q}$ for $i \in \{1, 2, \ldots, n_{max}\}$ and $\rho_i = 0$ otherwise). Then, the total excess resources that a forager can expect to collect if it occupies a patch in state i at time 0 are described by entry i of the vector $\boldsymbol{\phi}$, where

$$\boldsymbol{\phi} = \int_0^{\infty} \left(\mathbf{U}(t) - \mathbf{U}^*\right)^{\mathsf{T}} \boldsymbol{\rho}\, dt. \tag{S24}$$

Divergence of social learning phenotypes in the basic model

Consider a rare mutant with social learning phenotype s_m arising in a population that is otherwise monomorphic for the convergence-stable social learning phenotype s^*. Over time the mutant will become distributed according to $\mathbf{u^*}(s_m)$. Consider a randomly selected individual mutant that is confronted by a learning opportunity. Assume that after the learning event, the mutant will revert to the convergence-stable social learning phenotype. If the mutant expresses social learning phenotype s_m at the learning event, then after learning

and subsequent movement between patches, it will be distributed according to $\mathbf{L}(s_m)\mathbf{u}^*(s_m)$, and the expected resource gained due to the learning event will be $\mathbf{L}(s_m)\mathbf{u}^*(s_m) \cdot \boldsymbol{\phi}$. If the mutant expresses social learning phenotype s^* at the learning event, then after learning and any subsequent movement it will be distributed according to $\mathbf{L}(s^*)\mathbf{u}^*(s_m)$, and the expected resource gained due to the learning event will be $\mathbf{L}(s^*)\mathbf{u}^*(s_m) \cdot \boldsymbol{\phi}$. If $\mathbf{L}(s_m)\mathbf{u}^*(s_m) \cdot \boldsymbol{\phi} > \mathbf{L}(s^*)\mathbf{u}^*(s_m) \cdot \boldsymbol{\phi}$, then the mutant will benefit by expressing the mutant phenotype in the current learning event. Furthermore, by induction, the mutant will benefit by using the mutant phenotype at each future learning opportunity. Therefore, the condition for a mutant phenotype to invade a resident population monomorphic for the convergence-stable social learning phenotype, and thus the condition for the social learning phenotypes in the population to diverge, is

$$\left(\mathbf{L}(s_m) - \mathbf{L}(s^*)\right)\mathbf{u}^*(s_m) \cdot \boldsymbol{\phi} > 0. \tag{S25}$$

Further insight can be gained by rearranging inequality (S25). $\mathbf{L}_{\mathbf{sl}}\mathbf{u}^*(s^*) \cdot \boldsymbol{\phi}$ and $\mathbf{L}_{\mathbf{il}}\mathbf{u}^*(s^*) \cdot \boldsymbol{\phi}$ are the expected resource gains due to one social learning event or one individual learning event, respectively, for a resident forager with the convergence-stable social learning phenotype. Furthermore,

$$\mathbf{L}_{\mathbf{sl}}\mathbf{u}^*(s^*) \cdot \boldsymbol{\phi} = \mathbf{L}_{\mathbf{il}}\mathbf{u}^*(s^*) \cdot \boldsymbol{\phi}. \tag{S26}$$

Otherwise, selection would favour the frequency one learning type to increase, and s^* would not be convergence stable. Equation (S26) can be rearranged to

$$(\mathbf{L}_{\mathbf{sl}} - \mathbf{L}_{\mathbf{il}})\mathbf{u}^*(s^*) \cdot \boldsymbol{\phi} = 0. \tag{S27}$$

From equation (S22) we have

$$\mathbf{L}(s_m) - \mathbf{L}(s^*) = (s_m\mathbf{L}_{\mathbf{sl}} + (1 - s_m)\mathbf{L}_{\mathbf{il}}) - (s^*\mathbf{L}_{\mathbf{sl}} + (1 - s^*)\mathbf{L}_{\mathbf{il}}), \tag{S28}$$

which can be rearranged to

$$\mathbf{L}(s_m) - \mathbf{L}(s^*) = (s_m - s^*)(\mathbf{L}_{\mathbf{sl}} - \mathbf{L}_{\mathbf{il}}). \tag{S29}$$

By substitution into equation (S27) and multiplication by the scalar constant $(s_m - s^*)$, we can obtain

$$\left(\mathbf{L}(s_m) - \mathbf{L}(s^*)\right)\mathbf{u}^*(s^*) \cdot \boldsymbol{\phi} = 0. \tag{S30}$$

Therefore, we can rewrite inequality (S25) as

$$\left(\mathbf{L}(s_m) - \mathbf{L}(s^*)\right)\mathbf{u}^*(s_m) \cdot \boldsymbol{\phi} - \left(\mathbf{L}(s_m) - \mathbf{L}(s^*)\right)\mathbf{u}^*(s^*) \cdot \boldsymbol{\phi} > 0 \tag{S31}$$

and rearrange to obtain

$$\left(\left(\mathbf{L}(s_m) - \mathbf{L}(s^*)\right)^{\mathrm{T}}\boldsymbol{\phi}\right) \cdot \left(\mathbf{u}^*(s_m) - \mathbf{u}^*(s^*)\right) > 0. \tag{S32}$$

Inequality (S32) has a convenient interpretation. Entry *i* of the vector $\boldsymbol{\delta} = \left(\mathbf{u}^*(s_m)\text{-}\ \mathbf{u}^*(s^*)\right)$ describes how much the proportion of the mutant population exceeds the proportion of the resident population that occupies patches in state *i*. Entry *i* of the vector $\boldsymbol{\kappa} = \left(\left(\mathbf{L}(s_m) - \mathbf{L}(s^*)\right)^{\mathrm{T}}\boldsymbol{\phi}\right)$ describes the gain that a forager achieves by expressing the mutant phenotype rather than the resident phenotype when it occupies a patch in state *i*. If expressing the mutant phenotype tends to concentrate foragers in patches where it is advantageous to express the mutant phenotype again, then inequality (S32) will be true. The mutant phenotype will invade the resident, and we expect social learning phenotypes in the population to diverge.

Figure (S2) illustrates a system in which we expect social learning phenotypes to diverge, and figure (S3) illustrates a system with an evolutionarily stable social learning phenotype. In each figure, the *x*-axis represents good patches (white background) and bad patches (grey background) occupied by 1 to n_{max} foragers. Panel A shows **δ** and panel B shows **κ** for mutants with more (blue) and less (red) social learning than the convergence-stable phenotype. Panel C shows the cumulative sum of $\boldsymbol{\kappa} \circ \boldsymbol{\delta}$ for each mutant, where ∘ indicates the Hadamard product. Thus, in panel C, the value of the plotted functions at i is $\sum_{j=1}^{i} \kappa_j \delta_j$. The most important points in panel C are those in rightmost patch, which show $\boldsymbol{\kappa} \cdot \boldsymbol{\delta}$ for the two mutants. If these are both positive, then the social learning phenotypes in that population will diverge. If these are both negative, then the convergence-stable social learning phenotype is evolutionarily stable. The rest of the function is useful because it illustrates how the distribution of mutants among patches in different states either favours (where the functions slope upward) or disfavours (where the functions slope downward) the divergence of social learning phenotypes.

S4. Studying the evolutionary trajectories for the model extensions

For rules 1 and 2, the evolving traits are h and f, respectively. We define $s^{\bullet}$ as the proportion of all learning that is social learning in a monomorphic population at its steady-state distribution. For any system with given c and g, $s^{\bullet}$ decreases monotonically as h or f increase. Thus, for any value of $s^{\bullet}$ between 0 and 1, we can compute a unique corresponding value of h or f, and vice versa.

The proportion $s^{\bullet}$ is not a trait – it is an emergent property of the system. Nonetheless, it is convenient to use $s^{\bullet}$ to control the step sizes in our adaptive dynamics algorithm. This is true because, for some values of h and f, small changes in the value of the evolving trait have very little effect on forager behaviour. For example, when $f > n_{max} - 1$, foragers use social learning only if they are in fully occupied patches, but we set n_{max} so that foragers are rarely in fully-occupied patches. Similarly, when $f < 1$, foragers use individual learning only if they are alone in patches. But, when $f < 1$, individual learning is rare, and when individual learning is rare foragers are rarely alone. In each of these cases, small changes in f have almost no effect on forager behaviour. In contrast, when the value of f is intermediate, foragers may frequently occupy patches with exactly f_t foragers, and small changes in f can have large effects on forager behaviour. Using $s^{\bullet}$ to control the step size in the adaptive dynamics algorithm allows us to standardise the step size in terms of forager behaviour and facilitates the computation of convergence-stable states.

To study the evolution of social learning under rules 1 and 2, we studied potential invasions by mutant phenotypes as for the basic model, but substituting $s^{\cdot}$ for s. For each potential invasion we back-calculated the resident and mutant social learning phenotypes (*i.e.*, h or f) from the resident and mutant values of $s^{\cdot}$, we used those phenotypes to obtain $\mathbf{M}(\mathbf{v})$ and $\mathbf{N}$, and we calculated w_{res} and w_{mut} to test whether the invasion was successful. If the invasion was successful, we replaced the resident phenotype with the mutant. We iterated this process to find the convergence stable social learning phenotypes, and we tested the stability of these phenotypes as described for the basic model.

Under rule 3, the occupancy threshold for social learning evolves separately in good and bad patches, so the social learning phenotype has two dimensions. We defined $s_g^{\cdot}$ and $s_b^{\cdot}$ as the proportions of all learning that are social learning in good patches and bad patches, respectively, for a monomorphic population at its steady-state distribution. For any set of social learning frequencies $(s_g^{\cdot}, s_b^{\cdot})$, we can compute the unique pair of values of (f_g, f_b) that produce those frequencies. To study how f_g and f_b evolve, we initialised the model with a resident population with phenotype (f_{gr}, f_{br}) that produces social learning frequencies $(s_{gr}^{\cdot}, s_{br}^{\cdot})$. Then, we created four mutants to the resident phenotype with different steady-state social learning frequencies. We denote these mutants as g^+ (with social learning frequencies $(s_{gr}^{\cdot} + m, s_{br}^{\cdot})$), g^- (with $(s_{gr}^{\cdot} - m, s_{br}^{\cdot})$), b^+ (with $(s_{gr}^{\cdot}, s_{br}^{\cdot} + m)$) and b^- (with $(s_{gr}^{\cdot}, s_{br}^{\cdot} - m)$), where m is the evolutionary step size, which we set initially to 10^{-2}. From the social learning frequencies of each mutant, we back-calculated the mutant social

learning rules (f_{gm}, f_{bm}). We used (f_{gr}, f_{br}) to compute $\mathbf{M}(\mathbf{v})$ and solve for $\mathbf{v^*}$ for the resident population, and we used (f_{gm}, f_{bm}) to compute $\mathbf{N}$ and solve for $\mathbf{u^*}$ for each mutant phenotype. We used $\mathbf{v^*}$ and $\mathbf{u^*}$ to calculate the log relative fitness for each mutant in the resident population. We denote these log relative fitnesses with ω_{g+}, ω_{g-}, ω_{b+}, and ω_{b-}, where, for example, $\omega_{g+} = \ln(w_{g+}/w_{res})$. If any mutant could invade the resident population, then we created a new resident phenotype with steady-state social learning frequencies $\left(s^{\cdot}_{gr} + m_{gb}, s^{\cdot}_{br} + m_{bg}\right)$, where

$$m_{ij} = m\,(\omega_{i+} - \omega_{i-})\left((\omega_{i+} - \omega_{i-})^2 + \left(\omega_{j+} - \omega_{j-}\right)^2\right)^{-1/2}. \tag{S33}$$

Under equation (S33), the change in $s^{\cdot}_{gr}$ and $s^{\cdot}_{br}$ after replacement of the resident is proportional to the relative fitness of the mutations that affect $s^{\cdot}_{g}$ and $s^{\cdot}_{b}$. We iterated this process to simulate evolution.

The signs of m_{gb} and m_{bg} can be positive or negative, and thus the directions in which f_g and f_b evolve can change. If the signs of both m_{gb} and m_{bg} change in the same iteration, then our simulated evolutionary process is approaching a convergence-stable point. When this happened, then instead of replacing the resident with the mutant, we decreased the evolutionary step size m by a factor of $10^{1/2}$ and continued the simulation. We continued until we reached a set (f_{gr}, f_{br}) where no mutant could invade or until $m = 10^{-7}$, in which case we were close to a convergence stable point. We denote this point by $\left(f^*_g, f^*_b\right)$ and we denote the social learning frequencies it produces by $\left(s^*_g, s^*_b\right)$. Our algorithm approximates the evolutionary trajectory we would expect if i)

mutations that affect $s_g^{\cdot}$ and $s_b^{\cdot}$ are independent and occur with equal probability; ii) mutations are small, and rare enough that each new mutation either fixes or is eliminated before a new mutation arises; and iii) the probability that any mutation fixes is proportional to its relative fitness.

The evolutionary trajectory in our algorithm is obtained by studying small mutations that affect $s_g^{\cdot}$ and $s_b^{\cdot}$ independently, and it may not be the same trajectory we would obtain by studying small mutations that affect f_g and f_b independently. However, we are interested in the behaviour of the system at the convergence-stable phenotype, and if the convergence-stable phenotype is a global attractor, then the trajectory we use to reach that phenotype does not matter. We cannot prove that the convergence-stable phenotypes reached by our algorithm are always global attractors, but in spot-checks where we studied the same systems from different starting points, we always reached the same convergence-stable phenotypes.

To assess whether each convergence-stable phenotype $\left(f_g^*, f_b^*\right)$ obtained in our analysis is evolutionarily stable or is an evolutionary branching point, we tested for invasion by all mutants that produce social learning frequencies in the sets i) $(s_m^{\cdot}, s_b^*)$ and $\left(s_g^*, s_m^{\cdot}\right)$ for $s_m^{\cdot} \in \{0.01, 0.02, \dots, 1\}$ and ii) $\left(s_g^* + 0.01\cos(2\pi j/12), s_b^* + 0.01\sin(2\pi j/12)\right)$ for $j \in \{1, 2, \dots, 12\}$. Set i) tests whether any mutation that affects only $s_g^{\cdot}$ or $s_b^{\cdot}$ can invade the convergence-stable phenotype, and set ii) tests whether mutations with small effects on both $s_g^{\cdot}$ and $s_b^{\cdot}$ can invade the convergence-stable phenotype.

S5. Illustrating the mechanisms that favour the divergence of social learning phenotypes in model extensions 1 and 2

Preliminaries

Let $\mathbf{L_1}(h)$ and $\mathbf{L_2}(f)$ be transition matrices that describe the probability that a forager in a patch in state *j* moves to a patch in state *i* after a learning event in model extensions 1 and 2, respectively. Then, $\mathbf{L_1}(h)$ has entries

$$L_{1,ij}(h) = \begin{cases} L_{il,ij}, & j < \lfloor h \rfloor \\ (h - \lfloor h \rfloor)L_{il,ij} + (1 - h + \lfloor h \rfloor)L_{sl,ij}, & j = \lfloor h \rfloor \\ L_{sl,ij}, & j > \lfloor h \rfloor \end{cases} \tag{S34}$$

if $h \leq n_{max} + 1$ and

$$L_{1,ij}(h) = \begin{cases} L_{il,ij}, & j < \lfloor h \rfloor \\ (h - \lfloor h \rfloor)L_{il,ij} + (1 - h + \lfloor h \rfloor)L_{sl,ij}, & j \geq \lfloor h \rfloor \end{cases} \tag{S35}$$

otherwise, and $\mathbf{L_2}(f)$ has entries

$$L_{2,ij}(f) = \begin{cases} L_{il,ij}, & j < \lfloor f \rfloor \\ (f - \lfloor f \rfloor)L_{il,ij} + (1 - f + \lfloor f \rfloor)L_{sl,ij}, & j = \lfloor f \rfloor \\ L_{sl,ij}, & \lfloor f \rfloor < j \leq n_{max} + 1 \\ L_{il,ij}, & n_{max} + 1 < j < n_{max} + 1 + \lfloor f \rfloor \\ (f - \lfloor f \rfloor)L_{il,ij} + (1 - f + \lfloor f \rfloor)L_{sl,ij}, & j = n_{max} + 1 + \lfloor f \rfloor \\ L_{sl,ij}, & j > n_{max} + 1 + \lfloor f \rfloor \end{cases}. \tag{S36}$$

Divergence of social learning phenotypes in model extensions 1 and 2

As in the basic model, we expect social learning phenotypes to diverge if a forager that expresses a rare mutant phenotype can collect more resources than

a forager that expresses the convergence-stable phenotype. Substituting $\mathbf{L_j}$ for $\mathbf{L}$ in inequality (S25), the condition for the divergence of social learning phenotypes in model extension j is

$$\left(\mathbf{L_j}(x_m) - \mathbf{L_j}(x^*)\right) \mathbf{u}^{\mathbf{*}}(x_m) \cdot \boldsymbol{\phi} > 0, \tag{S36}$$

where x_m and x^* are the mutant and resident social learning thresholds (*i.e.*, h_m and h^* if j = 1, and f_m and f^* if j = 2) respectively. Entry i of vector $\boldsymbol{\gamma_j} = \left(\mathbf{L_j}(x_m) - \mathbf{L_j}(x^*)\right) \mathbf{u}^{\mathbf{*}}(x_m)$ describes the difference in the proportion of mutants in model extension j that occupy patches in state i immediately after a learning event in which they have expressed the mutant phenotype rather than the convergence-stable phenotype. If $\gamma_{ji} > 0$, then expressing the mutant phenotype concentrates mutants in patches in state i in model extension j. Entry i of $\boldsymbol{\phi}$ describes the long-term value of patch i. Thus, if expressing the mutant phenotype tends to concentrate foragers in more valuable patches, then the mutant phenotype is favoured to invade the resident population.

Figures (S4) and (S6) illustrate systems in which we expect social learning phenotypes to diverge, and figures (S5) and (S7) illustrate systems with evolutionarily stable social learning phenotypes, in model extensions 1 and 2 respectively. In each figure, the x-axis represents good patches (white background) and bad patches (grey background) occupied by 1 to n_{max} foragers. Panel A shows $\boldsymbol{\gamma}$ for mutants with more (blue) and less (red) social learning than the convergence-stable phenotype, and panel B shows $\boldsymbol{\phi}$. Panel C shows the cumulative sum of $\boldsymbol{\gamma} \circ \boldsymbol{\phi}$ for each mutant, where $\circ$ indicates the Hadamard

product. Thus, the value of the plotted functions at i is $\sum_{j=1}^{i} \gamma_j \phi_j$. The most important points in panel C are those in rightmost patch, which show $\boldsymbol{\gamma} \cdot \boldsymbol{\phi}$ for the two mutants. If these are both positive, then social learning phenotypes in the population will diverge. If these are both negative, then the convergence-stable social learning phenotype is also evolutionarily stable. The rest of the function is useful because it illustrates how the mutant phenotype concentrates foragers in patches with higher (where the functions slope upward) or lower (where the functions slope downward) long-term resource collection rates.

Because equation (S22) does not hold for the model extensions, we cannot obtain an analog for inequality (S32). Thus, for a mutant phenotype to invade a resident population with the convergence-stable social learning phenotype, it is neither necessary nor sufficient that expressing the mutant phenotype cause foragers to become concentrated in patches where it is advantageous to express that phenotype again.

Figure S1

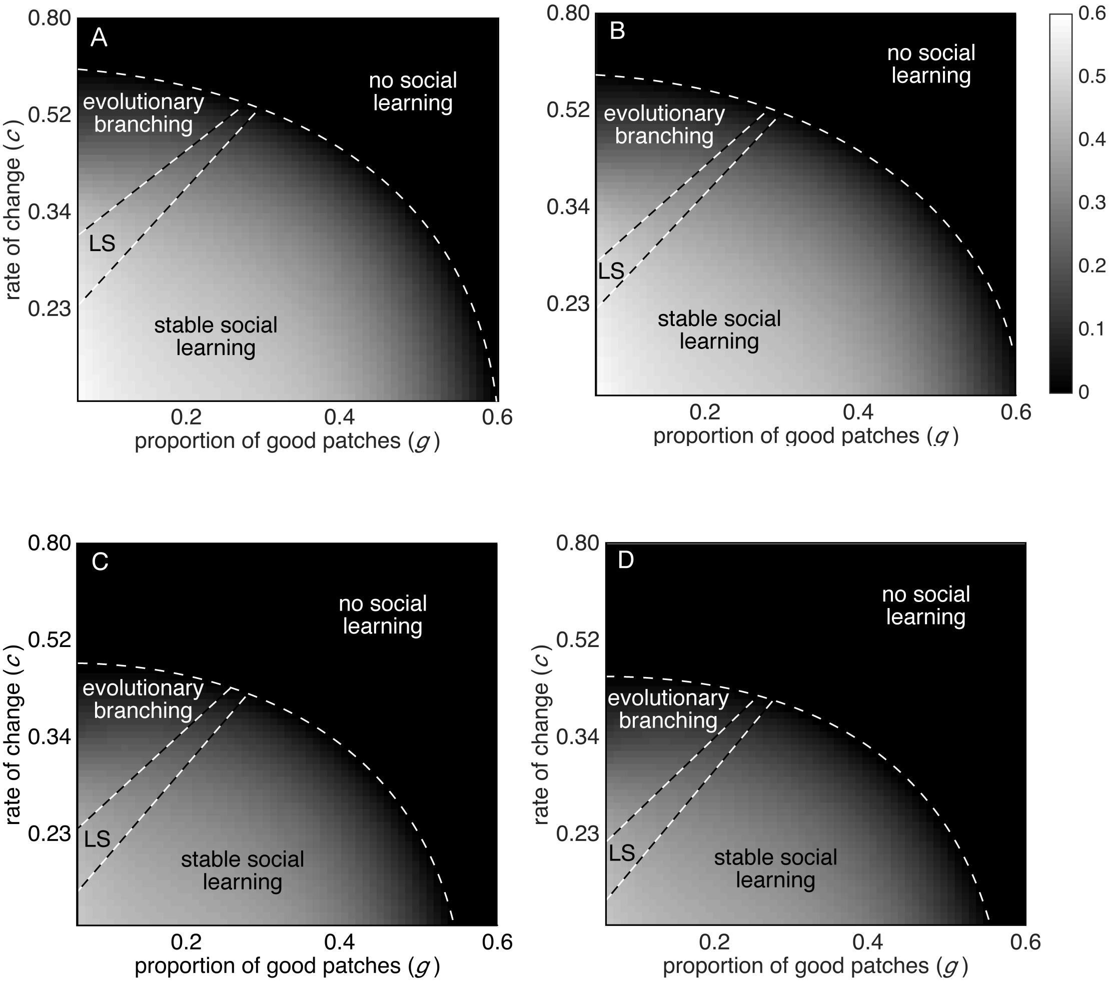

Figure S1. The proportion and evolutionary stability of social learning that evolves under different combinations of environmental heterogeneity, environmental change, competition strength (*q*) and population density (*D*). The competition strength was 0.9 (A, B) or 1.1 (C, D) and the population density was 0.9 (A, C) or 1.1 (B, D). LS indicates the part of parameter space where the evolved social learning phenotype is locally stable. Results are qualitatively similar to those presented in figure 1.

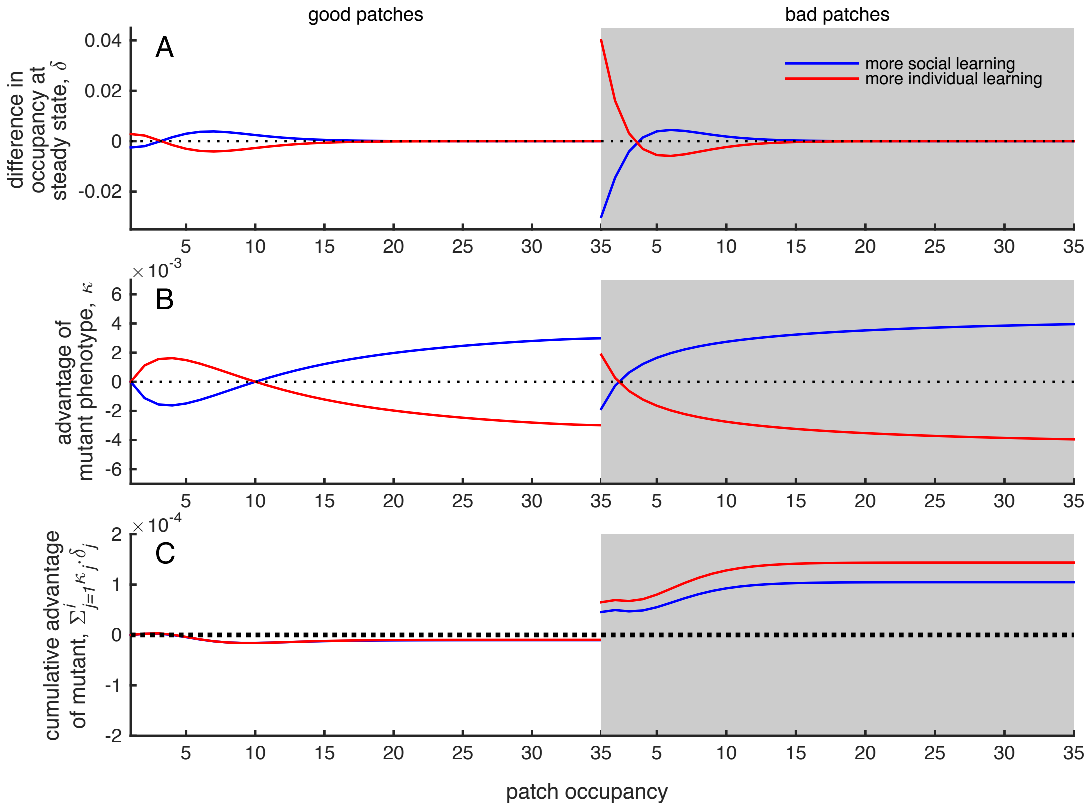

Figure S2. Illustration of a system where the divergence of social learning phenotypes is favoured (basic model, c = 0.45, g = 0.1, s^* = 0.12, $s_m \in$ {0.02 (red), 0.22 (blue)}). (A) shows the difference between the proportion of mutants and residents in patches in different states, and (B) shows the advantage foragers in each patch state achieve by using the mutant phenotype. Foragers with each mutant phenotype are relatively concentrated in patches where expressing that mutant phenotype is advantageous (C). Individual learning concentrates foragers in low occupancy patches where expressing individual learning is advantageous, and social learning concentrates foragers in moderate to high-occupancy patches where expressing social learning is advantageous.

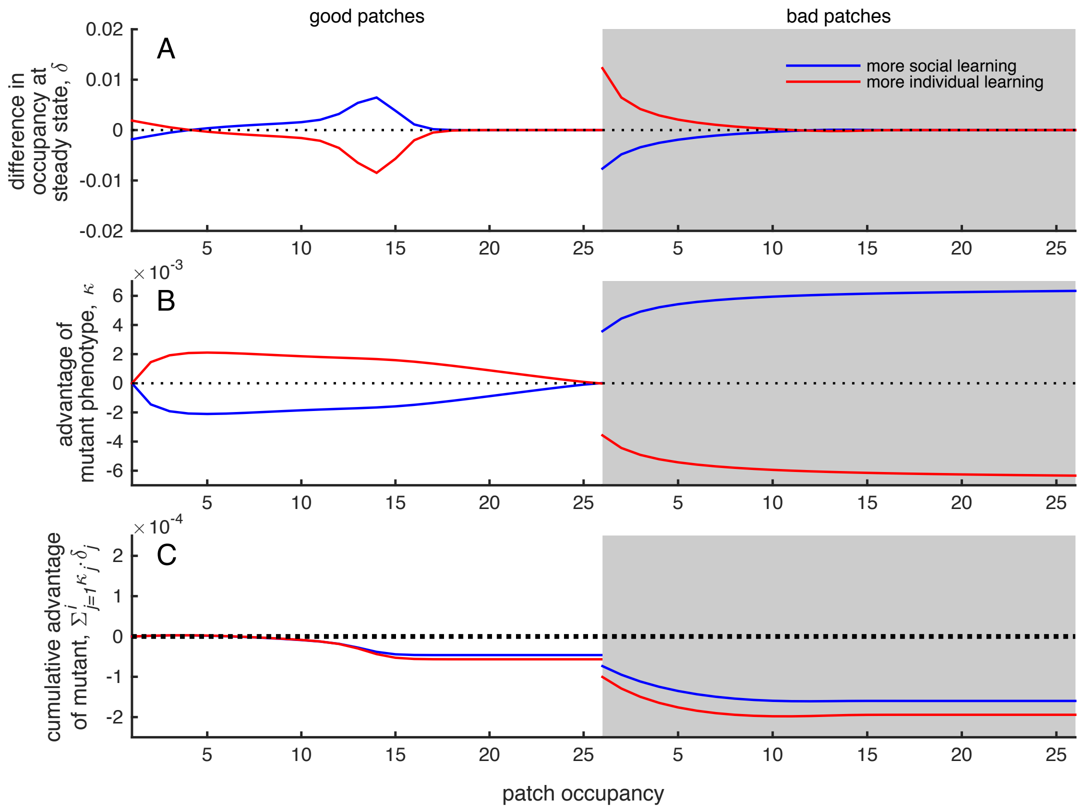

Figure S3. Illustration of a system where the convergence-stable social learning phenotype is evolutionarily stable (basic model, c = 0.15, g = 0.1, s^* = 0.48, $s_m \in$ {0.38 (red), 0.58 (blue)}). (A) shows the difference between the proportion of mutants and residents in patches in different states, and (B) shows the advantage foragers in each patch state achieve by using the mutant phenotype. As in figure (S2), foragers that express more individual learning are relatively more concentrated in low occupancy patches and in bad patches. Patch quality changes more slowly in this system, so at any point in time most good patches have already been found and are densely occupied. Social learning usually finds these densely occupied good patches, but rarely finds low-occupancy good patches. As a result, individual learning is favoured in high-occupancy good patches, which are most frequently discovered by social learning. Similarly, because social learning usually finds high-occupancy good patches, it is a reliable way for foragers in bad patches to begin collecting resources quickly. Thus, social learning is favoured in bad patches where phenotypes that use more individual learning are concentrated. As a result, it is better for foragers to change than to repeat their most recent learning type, and social learning phenotypes in the population do not diverge (C).

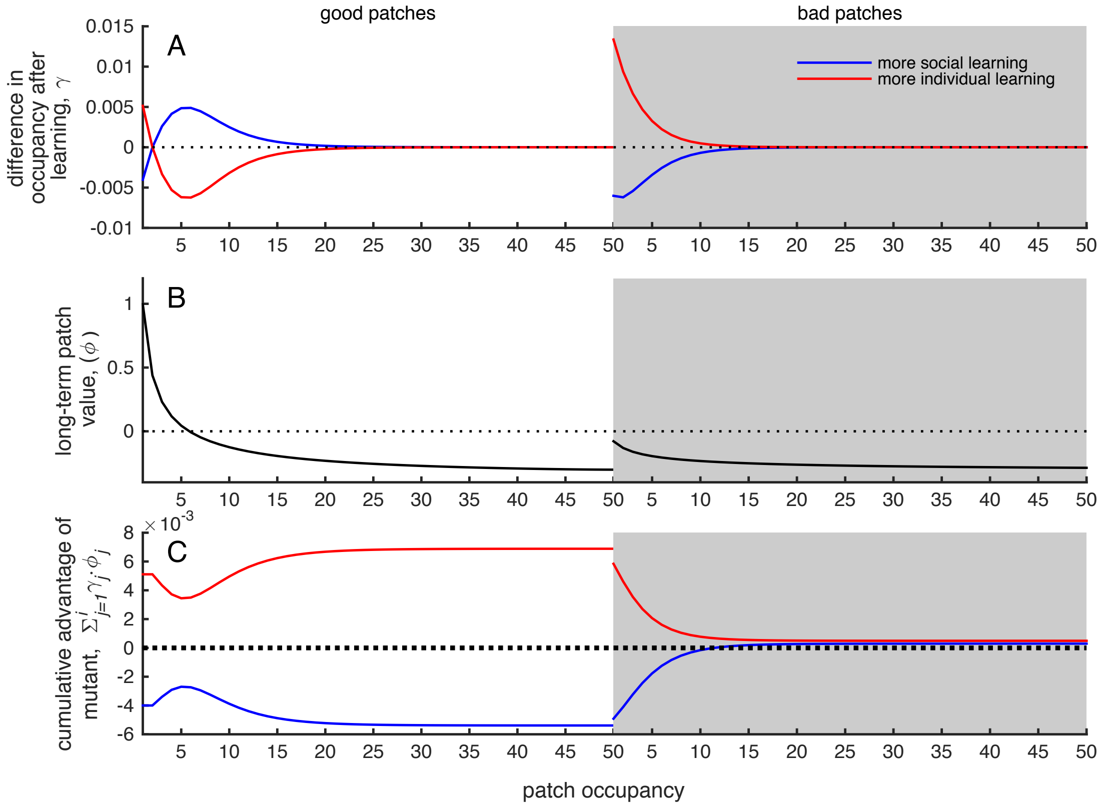

Figure S4. Illustration of a system where the divergence of social learning phenotypes is favoured (model extension 1, c = 0.4, g = 0.2, h^* = 51.54, $h_m \in$ {51.29 (blue), 51.78 (red)}). (A) shows the difference between the proportion of mutants in patches in different states immediately after a learning event in which they express either the mutant or the convergence-stable phenotype. (B) shows the long-term value of each patch state. Where the cumulative advantage functions in (C) slope upward, the mutant learning phenotype concentrates foragers in patches with better-than-average returns or helps foragers to avoid patches with worse-than-average returns.

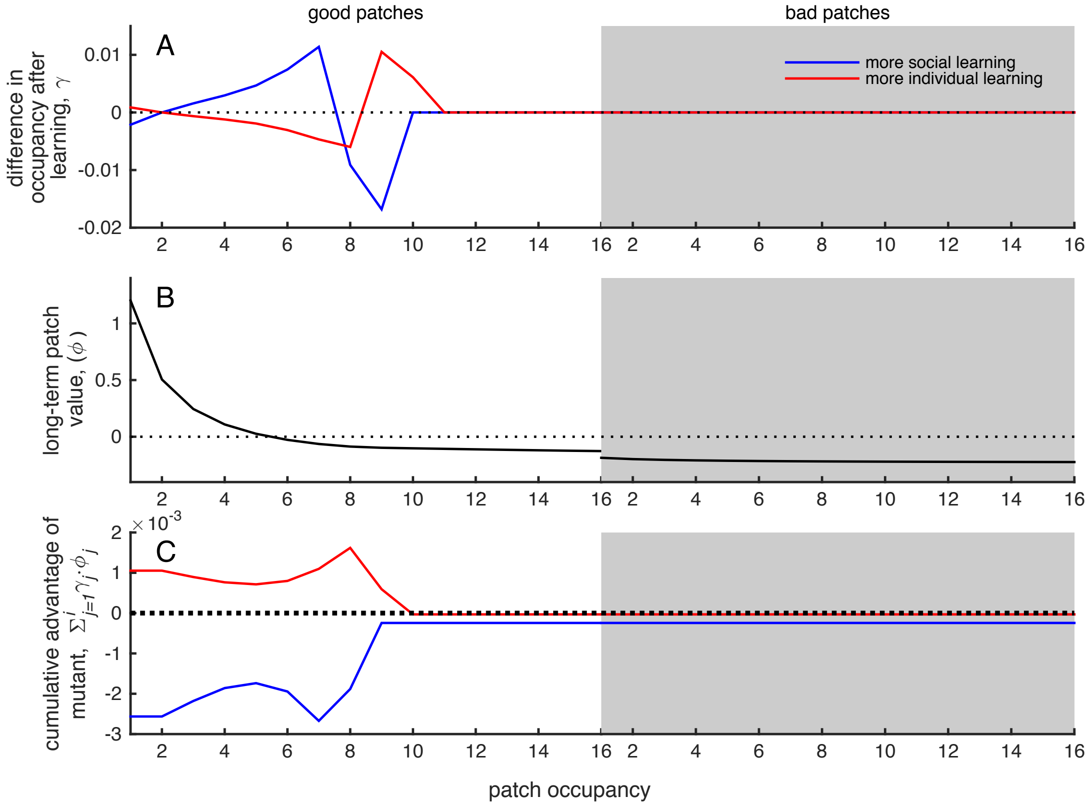

Figure S5. Illustration of a system where the convergence-stable social learning phenotype is evolutionarily stable (model extension 1, c = 0.15, g = 0.2, h^* = 9.64, $h_m \in$ {8.69 (blue), 10.88 (red)}). (A) shows the difference between the proportion of mutants in patches in different states immediately after a learning event in which they express either the mutant or the convergence-stable phenotype. (B) shows the long-term value of each patch state. Where the cumulative advantage functions in (C) slope downward, the mutant learning phenotype concentrates foragers in patches with worse-than-average returns or prevents foragers from finding patches with better-than-average returns.

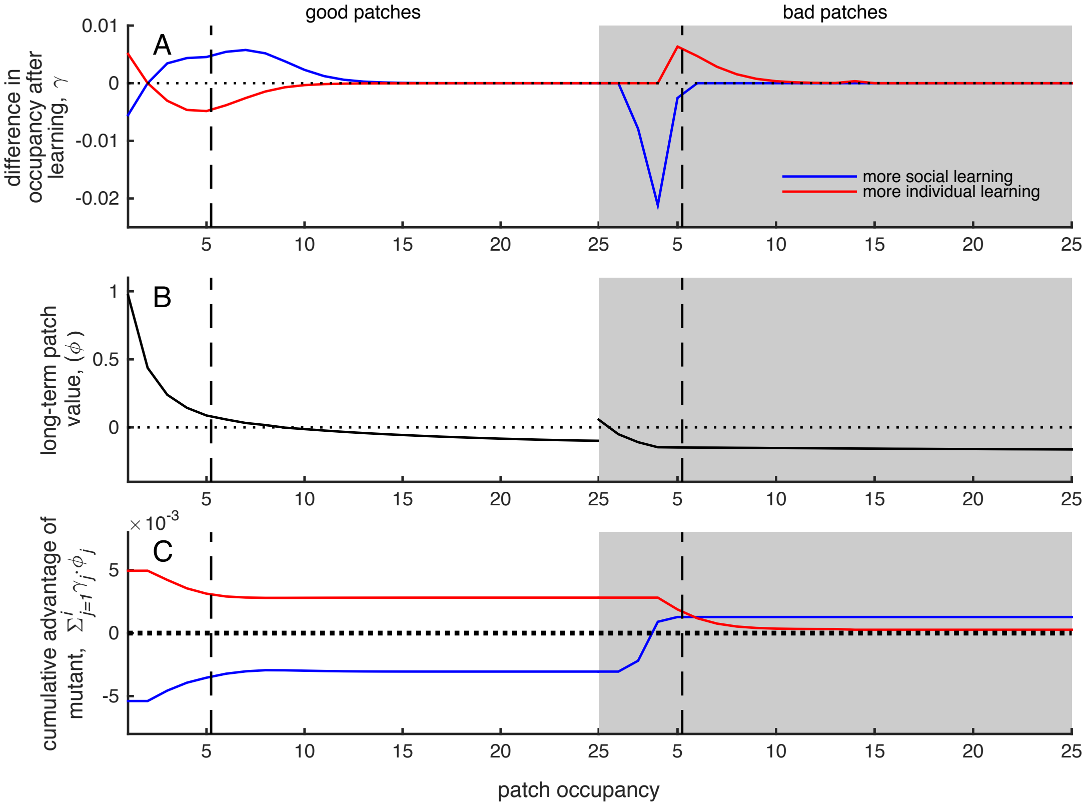

Figure S6. Illustration of a system where the divergence of social learning phenotypes is favoured (model extension 2, c = 0.5, g = 0.1, f* = 5.24, $f_m \in$ {3.63 (blue), 14.78 (red)}). Dashed vertical lines show the convergence-stable occupancy threshold f. (A) shows the difference between the proportion of mutants in patches in different states immediately after a learning event in which they express either the mutant or the convergence-stable phenotype. (B) shows the long-term value of each patch state. Where the cumulative advantage functions in (C) slope upward, the mutant learning phenotype concentrates foragers in patches with better-than-average returns or helps foragers to avoid patches with worse-than-average returns.

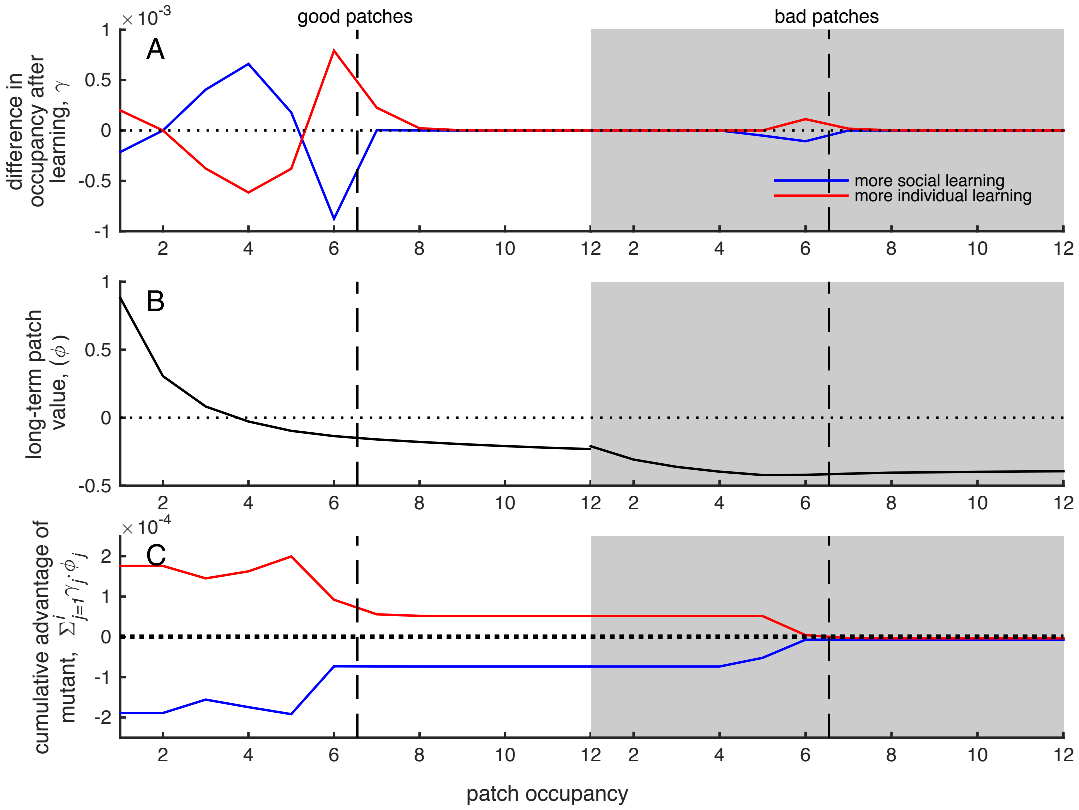

Figure S7. Illustration of a system where the convergence-stable social learning phenotype is evolutionarily stable (model extension 2, c = 0.4, g = 0.3, f^* = 6.55, $f_m \in$ {5.98 (blue), 13 (red)}). Dashed vertical lines show the convergence-stable occupancy threshold f. (A) shows the difference between the proportion of mutants in patches in different states immediately after a learning event in which they express either the mutant or the convergence-stable phenotype. (B) shows the long-term value of each patch state. Where the cumulative advantage functions in (C) slope downward, the mutant learning phenotype concentrates foragers in patches with worse-than-average returns or prevents foragers from finding patches with better-than-average returns.

	System in which social learning phenotypes diverge (figure S6)		System in which the convergence-stable social learning phenotype is evolutionary stable (figure S7)	
occupancy	good patches	bad patches	good patches	bad patches
1	0.0185	0.1691	0.0634	0.1398
2	0.0279	0.1702	0.1702	0.1365
3	0.0318	0.1501	0.2080	0.0844
4	0.0351	0.1274	0.1141	0.0309
5	0.0383	0.0440	0.0371	0.0074
6	0.0389	0.0265	0.0065	0.0007
7	0.0340	0.0168	0.0007	0.0000
8	0.0244	0.0094	0.0001	0.0000
9	0.0146	0.0047	0.0000	0.0000

Table S1. The proportion of all foragers with the convergence-stable social learning phenotype in good and bad patches with different occupancies in two examples of model extension 2. Columns 1 and 2 show the system illustrated in figure (S6), where the divergence of social learning phenotypes is favoured. Columns 3 and 4 show the system illustrated in figure (S7), where the convergence-stable social learning phenotype is evolutionarily stable. Highlighted cells show the patch occupancy at which foragers use a combination of social and individual learning. At lower occupancies they use only individual learning, and at higher occupancies they use only social learning.

In general, social learning is more advantageous in bad patches and individual learning is more advantageous in good patches. This is true because foragers in bad patches can improve their resource collection rate by finding any good patch, and social learning is more likely to find a good patch. In contrast, foragers in good patches can improve their resource collection rate only if they find less-occupied good patches, and individual learning is more likely to find less-occupied patches. In columns 1 and 2, there are more foragers in good patches above the occupancy threshold, and more foragers in bad patches below the occupancy threshold. If a mutant with more individual learning arises, that mutant has a higher threshold for social learning. This affects more foragers in good patches where individual learning is favoured than in bad patches where social learning is favoured, and as a result the mutant with more individual learning can invade. Similarly, if a mutant with more social learning arises, that mutant has a lower threshold for social learning. This affects more foragers in bad patches where social learning is favoured than in good patches where individual learning is favoured, and the mutant with more social learning can invade. In columns 3 and 4, the situation is different. Here, if a mutant with more social learning arises, the mutation affects more individuals in good patches where individual learning is favoured, and the mutant cannot invade.
